# Influence of the *IGFBP3*-202A/C Gene Polymorphism on Clinical Features and Surgery Outcome in Acromegalic Patients

**DOI:** 10.3389/fendo.2018.00751

**Published:** 2018-12-11

**Authors:** Ming Gao, Bin Zhu, Ping Li, Guojun Zhang, Kelin Chen, Hong Lv, Ruimin Ma, Limin Zhang, Yubo Fan, Xixiong Kang

**Affiliations:** ^1^Laboratory Diagnosis Center, Beijing Tiantan Hospital, Capital Medical University, Beijing, China; ^2^Beijing Engineering Research Center of Immunological Reagents and Clinical Research, Beijing, China; ^3^Department of Pharmacy, Beijing Tiantan Hospital, Capital Medical University, Beijing, China; ^4^Department of Endocrinology, Yuncheng Central Hospital, Shanxi Medical University, Yuncheng, China; ^5^Lab of Biological Science and Medical Engineering, Beihang University, Beijing, China

**Keywords:** acromegaly, *IGFBP3*-202A/C polymorphism, rs2854744, clinical feature, surgery outcome

## Abstract

**Purpose:** Excess growth hormone (GH) secretion in acromegaly patients results in increased levels of IGF-1 expression, which causes the clinical manifestations of acromegaly. IGF-1 levels are attenuated by IGFBP3, and a polymorphism in the promoter of IGFBP3 is known to affect the circulating level of IGFBP3 protein. The aim of the study was to evaluate the association of the *IGFBP3* gene polymorphism with clinical features and surgery outcomes in acromegaly. We also investigate the difference in *IGFBP3* polymorphism between acromegaly and general population.

**Methods:** The study included 102 acromegalic patients and 142 sex- and age-matched healthy controls. The genotyping of *IGFBP3* was carried out using the MassARRAY method. Patients were followed up for 4–12 months to estimate the neurosurgical effects. Clinical data were obtained from the medical records.

**Results:** The CC genotype, which is associated with decreased IGFBP3 levels, was less common in acromegaly patients than among the healthy controls; although, this correlation was not significant (*P* = 0.056). There was no association of the *IGFBP3* gene polymorphism with glucose, lipid, phosphorus, blood urea nitrogen, creatinine or uric acid levels. Additionally, there was no association between tumor size, texture, or hemorrhage/cyst, except there was a trend that more patients with the C allele (*P* = 0.054) needed additional treatment post-operation than did patients carrying the A allele (OR 1.985, 95% CI 0.983–4.008). Moreover, higher IGF-1 values after treatment were observed in patients carrying the C allele (*P* = 0.012 and *P* = 0.014 according to the additive model and dominant model, respectively).

**Conclusion:** Polymorphisms in *IGFBP3* may not influence metabolic parameters or pituitary tumor characteristics in acromegalic patients, but they may be associated with the hormone levels and surgery effects.

## Introduction

Acromegaly is a rare, chronic disorder caused by the endogenous excess of growth hormone (GH), which is most often secreted by a pituitary adenoma. The consequent hypersecretion of insulin-like growth factor-1 (IGF-1, a GH-induced liver protein) accounts for most clinical manifestations and treatment outcomes (1). Initial testing relies on the determination of GH and IGF-1 serum values. The 75 g oral glucose tolerance test (OGTT) with a nadir GH measurement is recognized as the gold standard diagnostic test. The therapeutic options for acromegaly are surgery (preferred), medical treatment, and radiotherapy (subsidiary).

Surgery to remove the pituitary adenoma is generally the first-line therapy for acromegaly with the goal of normalizing the GH and IGF-1 levels (2). However, adenomas in acromegaly are generally macroadenomas, and many of these tumors possess a hard texture or invade surrounding structures. Thus, total resection cannot always be achieved. Hence, the hormone levels in these cases decrease after partial tumor removal, but do not become normal (3). At this time, several treatment modalities, including medical therapy, radiation, and repeat surgery, are needed to control hormone secretion and tumor proliferation. Therefore, measuring serum GH and IGF-1 concentrations pre- and post-operation are important (4).

Recently, a number of novel genetic alterations have been identified that predispose individuals to pituitary adenomas. Zhang et al. reported that germline mutations in *CDH23* are associated with both familial and sporadic pituitary adenomas (5). Ye et al. performed large Genome-Wide Association Studies (GWAS) and showed that common variants at 10p12.31, 10q21.1, and 13q12.13 are correlated with sporadic pituitary adenoma in a Chinese Han population (6). Pituitary adenoma patients who have *AIP* or *GPR101* mutations often present with pituitary acromegaly or gigantism. *AIP* mutation carriers are younger at diagnosis and prone to macroadenoma (7–9).

IGF-1 and IGF-2 are growth factors that regulate cell proliferation, apoptosis, transformation, and differentiation (10). More than 75% of circulating IGFs is bound to Insulin-like growth factor binding proteins (IGFBPs), primarily IGFBP3, which forms a ternary complex. As the major circulating subtype of IGFBPs, IGFBP-3 competes with cell surface receptors for free IGF-1 and IGF-2, thereby controls bioavailability and half-life period of IGFs to the target tissues (11). Independent from its capacity of binding with IGFs, IGFBP-3 has its own effects including regulation of cell proliferation and induction of apoptosis (12). Several basic and clinical studies have revealed that IGFBP3 participates in the process of tumorigenesis (13–15). *IGFBP3* polymorphism rs2854744, located 202 bp upstream of the transcription start site (-202A/C), was reported to correlate with its promotor activity, and protein levels. The circulating level of IGFBP3 protein is correlated with the number of C alleles at this site, such that people who are CC have the least amount of circulating IGFBP3 and people who are AA have the most. In addition, the single nucleotide polymorphism (SNP) was correlated with hormonal activity and comorbidities of acromegaly (16–18). However, few studies have assessed the correlation of rs2854744 with the clinical characteristics of acromegalic patients, and the findings are inconsistent. To our knowledge, the association between the polymorphism and tumor size, texture, hemorrhage, and surgical outcome has not been studied. Thus, we tried to assess the impact of *IGFBP3* rs2854744 on sporadic acromegalic patients' clinical phenotype, laboratory measurements, and surgical effect in a Chinese Han population. The study also recruit 142 healthy controls, and the second aim of the study was to compare the incidence of the IGFBP3 polymorphism in patients with acromegaly vs the general population.

## Subjects and Methods

### Subjects

A cohort of 102 unrelated acromegaly patients (54 men, 48 women, mean age was 42.18 ± 10.28 years) were enrolled in the study from inpatients in Beijing Tiantan Hospital in 2017. Acromegaly was diagnosed based on the Endocrine Society's clinical practice guidelines (19). Patients underwent physical examinations, laboratory measurements, and pituitary contrast-enhanced magnetic resonance imaging (MRI) at diagnosis. This information was obtained from medical records. All patients had received neurosurgical treatment and were confirmed to have GH-secreting pituitary adenomas.

The study also included 142 healthy controls (69 men, 73 women, mean age 42.24 ± 11.63 years) to evaluate whether the allele frequency distribution in acromegaly was different from that of the general population. Participants were informed about the purpose of the protocol with signed consent forms. The protocol was approved by the Ethics Committee of Beijing Tiantan Hospital.

### Division Into Groups

Patients were followed up 4–12 months in the outpatient and inpatient ward. Sometimes telephone calls were made to obtain up-to-date follow-up data for patients who did not make subsequent visits to our hospital. For analysis purposes, acromegaly was divided into various subgroups by different classification methods: patients who only received surgery were grouped into monotherapy groups. Those who underwent other treatment during the follow-up period, including reoperation, somatostatin analogs, and adjuvant radiotherapy, were grouped into combined therapy groups. According to tumor sizes, pituitary adenomas were classified into microadenomas (largest diameter ≤10 mm) and macroadenomas (largest diameter >10 mm). If there were signs of hemorrhage/cysts on imaging, in the surgery records, and postoperation pathology, patients were categorized into two subgroups: apoplexy/cyst and non-apoplexy/cyst. According to the description of the tumor texture in the operation notes, patients were divided into soft tumor and hard tumor groups.

### Biochemical and Hormonal Assays

The following parameters were measured: GH, IGF-1, and IGFBP3 levels at diagnosis (baseline) and after surgery in hormonal analysis, fasting plasma glucose (FPG), lipid profile [total cholesterol (TC), triglycerides (TG), high density lipoprotein cholesterol (HDL-C), low density lipoprotein cholesterol (LDL-C)], and phosphorus (P) at baseline in metabolic analysis, and kidney indicators [blood urea nitrogen (BUN), creatinine (Cr), and uric acid (UA)] in renal function analysis. Since normal IGF-1 values vary among different ages and genders, the serum IGF-1 level was compared to reference values for age and gender. The IGF-1 index was calculated by dividing the IGF-I level by the upper limit of the normal value for age and gender (20). We used the common geometric formula, which is 1/2 (length × width × height) on MRI, to estimate tumor volumes (21).

### DNA Isolation and Genotyping

Blood samples from each participant were collected in tubes containing ethylene diamine tetraacetic acid (EDTA) and stored at −80°C until use. Genomic DNA was extracted from peripheral white blood cells using a Qiagen DNA purification kit (Qiagen, Hilden, Germany). The DNA quantity was determined using a NanoDrop 2000 spectrophotometer (Thermo Fisher, Waltham, MA, USA). Polymerase chain reaction (PCR) primer pairs were used to amplify rs2854744 in *IGFBP3*: GGTTCTTGTAGACGACAAGG (forward primer) and GTGCAGCTCGAGACTCGCC (reverse primer). Genotyping analysis of the included population was performed using time-of-flight mass spectrometry on a MassARRAY iPLEX platform (Sequenom, San Diego, CA, USA) in Bio Miao Biological Technology (Beijing).

### Statistical Analysis

Categorical variables are presented as the absolute count and percentage. After testing for normality, continuous variables were shown as the mean ± SD and median (interquartile range) for normally and nonnormally distributed parameters, respectively. The Chi-square test or Fisher's exact test was used to evaluate categorical variables. Odds ratios (ORs) with 95% CIs were calculated. To analyze differences in continuous variables among *IGFBP3* genotypes (AA, AC, CC genotypes), one-way ANOVA for normally distributed variables and a Kruskal–Wallis test for parameters with skewed distribution were performed. For the comparison of continuous variables between the AA group and C allele carriers (AC+CC), Student's *t-*test or the Mann–Whitney *U*-test were used according to the normality of distribution. Hardy-Weinberg equilibrium (HWE) was assessed by the χ^2^ test and a *P* value >0.05 was considered equilibrium. Two-tailed *P* < 0.05 was considered statistically significant. All statistical analyses were conducted with SPSS version 20.0 (SPSS Inc., Chicago, USA).

## Results

Genotypes of the *IGFBP3*-202A/C polymorphism (rs2854744) were available in 100 acromegalic patients, and the AA, AC, and CC genotypes were distributed as follows: 63 (*n* = 63), 33 (*n* = 33), and 4% (*n* = 4), respectively. On the other hand, there were 142 sex- and age-matched control subjects in the study to compare the distribution of the *IGFBP3* genotype frequencies between the acromegalic patients and the general population. 72 controls (50.7%) were homozygotes for the A allele (AA), 54 controls (38%) were heterozygotes (AC), and 16 controls (11.3%) were homozygotes for the C allele (CC). The observed genotype distribution was in Hardy–Weinberg equilibrium in the controls (χ^2^ = 1.4, *P* = 0.236), and cases (χ^2^ = 0.02, *P* = 0.9), which indicates the samples being studied can represent their groups. On the other hand, the IGFBP3-202A/C genotype among patients with acromegaly vs. controls had trend toward statistically significant difference (*P* = 0.056, Table 1).

**Table 1:** Analysis of the *IGFBP3* rs2854744 genotype distribution in acromegalic patients and controls.

**SNP**	**Genotype**	**Case (%) *N* = 100**	**Control (%) *N* = 142**	***P***	**Hardy-weinberg test**
rs2854744	AA	63 (63)	72 (50.7)	**0.056**	$\chi_{\text{control}}^{2}$ = 1.4
	AC	33 (33)	54 (38)		P = 0.236
	CC	4 (4)	16 (11.3)	

SNP, single nucleotide polymorphism. Bold value represents P value that is close to 0.05

In the 100 acromegaly patients, 65 patients (65%) were in the monotherapy group, 33 patients (33%) were in the combination therapy group, and 2 patients (2%) were lost to follow-up. According to available imaging and surgical records, 76 patients (84.4%) harbored macroadenomas, and 14 patients (15.6%) harbored microadenomas. There were 15 tumors (16.3%) that showed evidence of hemorrhage or cyst and 77 tumors (83.7%) without apoplexy/cyst. Sixty-four tumors (69.6%) had a soft texture, and 28 tumors (30.4%) had a hard or medium texture. The genotype and allele frequency of rs2584744 in each group are shown in Table 2. We did not find any significant differences in genotype frequency between subgroups regarding pituitary adenoma properties or therapeutic effect. However, we found a trend (Table 2, *P* = 0.054) that the presence of the C allele at the −202 site was associated with a greater chance for patients to receive additional treatment postoperation (OR 1.985, 95% CI 0.983–4.008) than that for patients carrying the A allele.

**Table 2:** Association between the *IGFBP3* rs2854744 polymorphism and clinical features of acromegalic patients.

**Classification**	**Genotype** ***N*** **(%)**			***P*^a^**	**Allele** ***N*** **(%)**		***P*^b^**	**OR (95% CI)**
	**AA**	**AC**	**CC**		**C**	**A**	
**THERAPEUTIC METHOD**								
Combination therapy	17 (51.5)	13 (39.4)	3 (9.1)	0.107	19 (28.8)	47 (71.2)	**0.054**	1.985 (0.983–4.008)
Monotherapy	44 (67.7)	20 (30.8)	1 (1.5)		22 (16.9)	108 (83.1)	
**TUMOR SIZE**								
Microadenoma	8 (57.1)	6 (42.9)	0 (0)	0.504	6 (21.4)	22 (78.6)	0.837	1.11 (0.413–2.976)
Macroadenoma	50 (65.8)	22 (28.9)	4 (5.3)		30 (19.7)	122 (80.3)	
**APOPLEXY/CYST**								
Yes	12 (80)	3 (20)	0 (0)	0.385	3 (10)	27 (90)	0.131	0.39 (0.112–1.372)
No	47 (61)	26 (33.8)	4 (5.2)		34 (22.1)	120 (77.9)	
**TUMOR TEXTURE**								
Hard	18 (64.3)	9 (32.1)	1 (3.6)	1	11 (19.6)	45 (80.4)	0.823	0.91 (0.417–2.003)
Soft	40 (62.5)	21 (32.8)	3 (4.7)		27 (21.1)	101 (78.9)	

a *Comparison among genotype AA, AC, and CC*.

b *Comparison between allele A and C. Bold value represents P value that is close to 0.05*.

**Figure 1:**
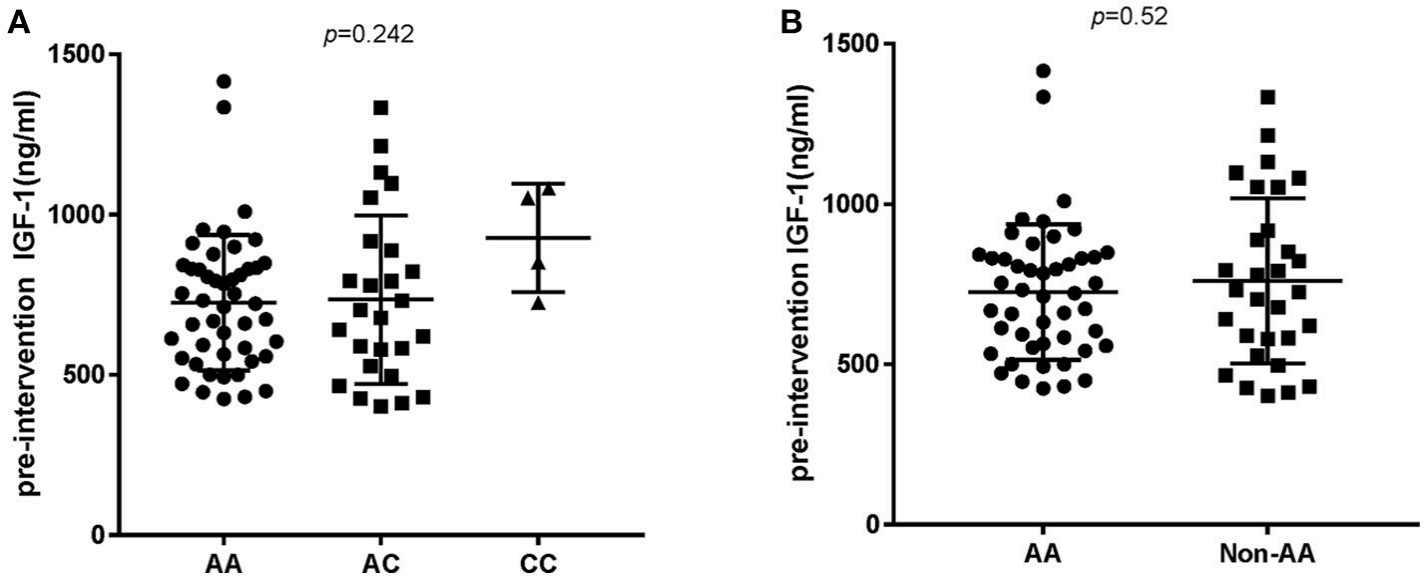
Pre-intervention IGF-1 level according to the *IGFBP3* genotype. **(A)** According to AA/AC/CC genotypes and **(B)** according to AA or Non-AA genotypes. No significant differences in IGF-1 baseline levels were observed.

**Figure 2:**
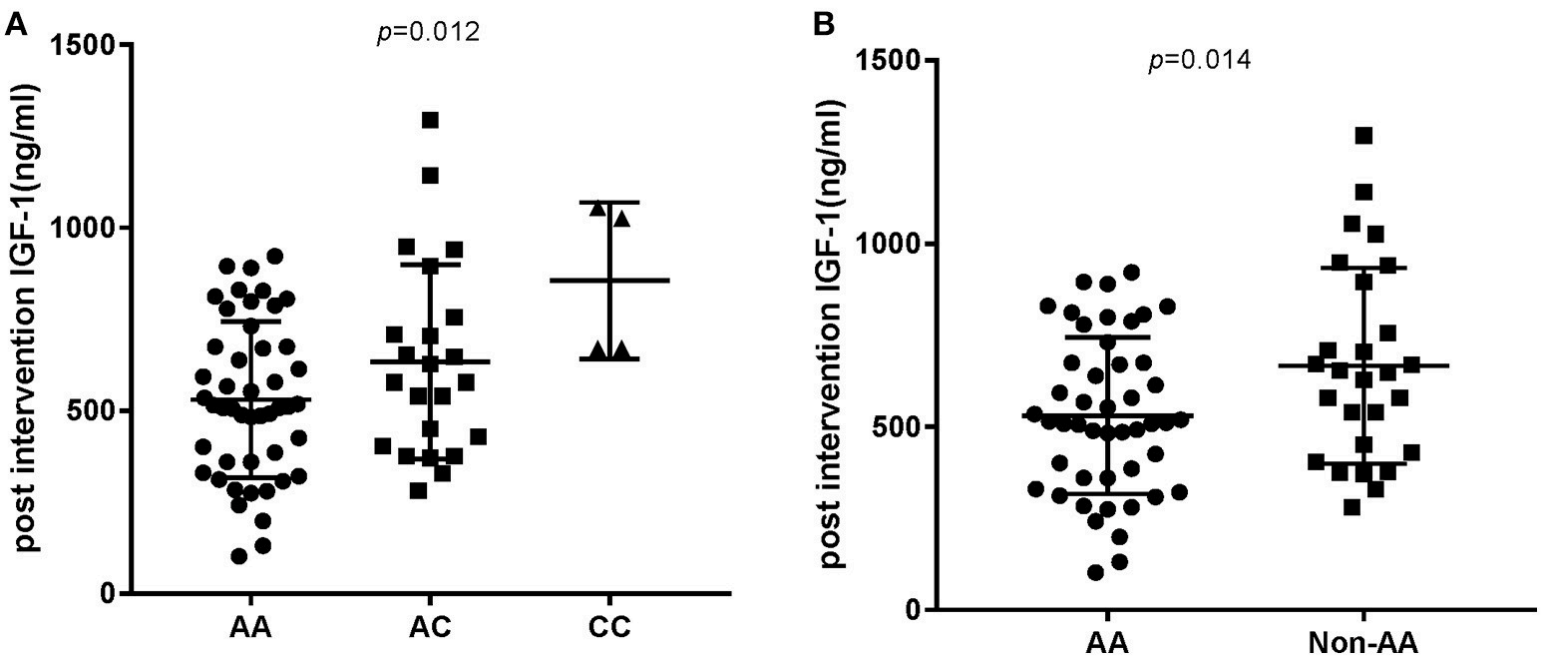
Post intervention IGF-1 level according to the *IGFBP3* genotype. **(A)** Post intervention IGF-1 level according to AA/AC/CC genotypes. *P*-value for comparison among the three groups was *p* = 0.012. **(B)** *P*-value for comparison between the AA and Non-AA genotypes was 0.014.

In order to analyze the association between the SNP and acromegaly laboratory measurements, patients were compared in three or two groups in accordance with *IGFBP3* genotypes. Due to the small number of patients with the CC allele (*n* = 4), patients who were C allele carriers for the *IGFBP3* genotype (AC+CC) were subgrouped and compared to those in the AA group. The results showed that there was no significant difference in the mean concentrations of glucose, lipid, or phosphorus levels at diagnosis according to the three genotypes (AA vs. AC vs. CC, additive model) as well as the two genotypes (AA vs. AC+CC, dominant model). Kidney indicators (BUN, Cr, and UA) among these genotypes did not display any significant difference either (Table 3). With regard to hormonal activities, higher IGF-1 values after treatment were observed in patients carrying the C allele (Table 4, *P* = 0.012 and *P* = 0.014, according to the additive and dominant model, respectively). Additionally, the IGF-1 index after treatment displayed similar differences according to specific genotypes (*P* = 0.01 and *P* = 0.042, according to the additive model and dominant model, respectively). Figures 1, 2 showing the distribution of pre- and post- intervention IGF-1 levels per genotype.

**Table 3:** Comparisons of the metabolic indicators of acromegalic patients in accordance with the *IGFBP3* rs2854744 polymorphism at diagnosis.

**Parameters**	**AA**	**AC**	**CC**	**AC+CC**	***P*^a^**	***P*^b^**
TG (mmol/L)	1.3 (0.98, 2.52)	1.09 (0.9, 1.45)	1.54 (1.2, 2.0)	1.1 (0.9, 1.54)	0.761	0.488
TC (mmol/L)	4.54 ± 1.04	4.57 ± 0.95	4.17 ± 0.87	4.51 ± 0.93	0.812	0.931
HDL-C (mmol/L)	1.3 ± 0.34	1.26 ± 0.23	1.27 ± 0.98	1.26 ± 0.21	0.875	0.654
LDL-C (mmol/L)	2.73 ± 0.85	2.74 ± 0.88	2.55 ± 0.7	2.71 ± 0.84	0.932	0.945
FPG (mmol/L)	5.3 (4.81, 6.14)	5.52 (5.12, 6.38)	5.73 (4.79, 6.28)	5.52 (5.12, 6.35)	0.394	0.201
BUN (mmol/L)	4.7 ± 1.34	4.28 ± 1.14	4.1 ± 0.75	4.26 ± 1.1	0.238	0.093
Cr (umol/L)	48.73 ± 13	49.46 ± 13.8	51.63 ± 15.94	49.69 ± 13.8	0.898	0.727
UA (umol/L)	310.1 ± 94.5	290.8 ± 77.7	330 ± 167.6	295.17 ± 87.5	0.63	0.503
P (mmol/L)	1.44 ± 0.26	1.42 ± 0.21	1.54 ± 0.18	1.43 ± 0.21	0.735	0.861

*Normal values are expressed as the mean ± SD, while nonnormally distributed parameters are expressed as the median (interquartile range)*.

*TG, triglycerides; TC, total cholesterol; HDL-C, high-density lipoprotein-cholesterol; LDL-C, low-density lipoprotein-cholesterol; FPG, fasting plasma glucose; BUN, blood urea nitrogen; Cr, creatinine; UA, uric acid; P, phosphorus*.

a *Additive model: AA vs. AC vs. CC*.

b *Dominant model: AA vs. (AC + CC)*.

**Table 4:** Comparisons of clinical and hormonal characteristics of acromegalic patients in accordance with the *IGFBP3* rs2854744 polymorphism at diagnosis and after treatment.

**Parameters**	**AA**	**AC**	**CC**	**AC+CC**	***P*^a^**	***P*^b^**
Volume (cm^3^)	2.43 (1.1, 7.12)	1.96 (0.72, 4.28)	1.97 (1.35, 4.34)	1.96 (0.75, 4.28)	0.41	0.186
**AT DIAGNOSIS**						
GH (ng/ml)	14.2 (6.42, 38.8)	12.7 (6.6, 33.2)	30.65 (8.9, 40)	17.05 (6.6, 38.5)	0.68	0.784
nGH (ng/ml)	8.9 (3.66, 18.6)			19.2 (3.67, 34)		0.288
IGF-1 (ng/ml)	725.17 ± 211	734.31 ± 262.4	927 ± 169.4	760 ± 258.4	0.242	0.52
IGF-1 index	2.65 ± 0.65	2.7 ± 0.87	3.5 ± 0.62	2.81 ± 0.87	0.083	0.35
IGFBP3 (ug/ml)	7.46 ± 1.24			6.98 ± 1.06		0.331
**AFTER TREATMENT**						
GH (ng/ml)	2.15 (0.77, 5.94)	3.53 (1.01, 6.14)	7.25 (1.67, 19.4)	3.91 (1.02, 6.69)	0.542	0.336
nGH (ng/ml)	2.15 (0.65, 10.45)			2.31 (1.1, 5.23)		0.803
IGF-1 (ng/ml)	519.2 ± 227.2	633.7 ± 265.7	855.5 ± 213.9	666.5 ± 267.4	**0.012**	**0.014**
IGF-1 index	1.95 ± 0.86	2.26 ± 0.88	3.48 ± 0.73	2.4 ± 0.93	**0.01**	**0.042**
IGFBP3 (ug/ml)	6.43 ± 0.95			5.73 ± 0.88		0.087

Normal values are expressed as the mean ± SD, while nonnormally distributed parameters are expressed as the median (interquartile range). Bold values mean P < 0.05.

*nGH, nadir GH*.

a *Additive model: AA vs. AC vs. CC*.

b *Dominant model: AA vs. (AC+CC)*.

## Discussion

In this study, we found that the *IGFBP3*-202A/C genotype distribution have a trend toward significantly different rates between patients and controls. We further evaluated their impacts on clinical features and the response to treatment in a large cohort of patients. This is the first study to focus on the effects of the *IGFBP3* polymorphism on pituitary tumor characteristics and surgical outcome in Chinese acromegalic patients.

In the present study, the distribution of the three *IGFBP3*-202A/C genetic variants was 63 AA, 33 AC, and 4% CC, which was similar to that observed in Spanish acromegaly patients(18). However, in Brazilian and Turkish acromegaly patients, AC heterozygosis was the predominant genotype, and the CC genotype was slightly more common than was the AA genotype (16, 17). Because the frequency of genotypes varies among different populations and ethnicities, several studies focused on the association of *IGFBP3* with acromegaly, obtaining different results. Ramos et al. demonstrated a strong association of the rs2854744 C allele with a higher baseline IGF-1 and a higher prevalence of cancer and polyps in a Spanish population (18). Jallad et al. observed an increased GH concentration after treatment in patients carrying the C allele (17). Akin et al. found no association of *IGFBP3*-202A/C polymorphisms with the clinical characteristics of acromegaly in a Turkish population (16).

Comparing among the clinical subgroups, there was a trend toward a difference in the allelic frequency between the monotherapy and the combination therapy group. Patients in the latter group had a higher frequency of the C allele, which means that operative patients with the C allele have a greater likelihood of needing additional treatment after surgery to control disease. We also examined various relationships between the *IGFBP3* genotypes and glucose, lipid, phosphorus, kidney index, tumor volume, plasma GH level, and plasma IGF-1 before and after surgery in patients with acromegaly. The results showed that the *IGFBP3* gene polymorphism was not associated with metabolic indicators of acromegaly, but had an effect on the hormone levels. Patients carrying the C allele correlated with higher IGF-1 levels after surgery, which is partially consistent with that reported in earlier studies and may explain the phenomenon that patients with the C allele need additional treatment postoperatively.

It has been reported that *IGFBP3*-202A/C variances can affect promoter activity and IGFBP3 levels. Several studies performed in healthy controls, adult GH-deficient patients, and in patients with cancer (22–25) showed a higher promoter activity (and higher IGFBP3 circulating levels) in AA>AC>CC genotypes. Our study had similar results. The serum IGFBP3 level was 7.404 ± 1.1 μg/mL in the AA genotype and 6.989 ± 1.065 μg/mL in the non-AA genotype, although the difference was not statistically significant (*P* = 0.331).

IGFBP3 is the major transport protein for IGF-1, modulating its half-life and biological activities. Some cell culture studies showed that IGFBP3 plays a vital role in cell survival or apoptosis in various microenvironments; meanwhile, several clinical studies have indicated that variations in the *IGFBP-3* genotype were associated with acromegaly risk and clinical features. Previous studies and our results suggest that patients with C alleles were correlated with lower IGFBP3 levels, showing increased IGF-1 activity and increased severity of the condition. Ultimately, the patients carrying the C allele had a higher IGF-1 and a greater possibility of receiving combined treatment.

There are some limitations in the present study. First, as the mechanism of acromegaly is dramatically complex and we only concentrated on the *IGFBP3* polymorphism, this result cannot completely explain the clinical phenotype. Second, some indexes in our research were nearly at the edge of the significance. Owing to the limited quantity of enrolled patients, we didn't add a sample size calculation to verify the present results. Nonetheless, we consider that our findings provided relevant clinical significance, as knowledge of the patients' specific polymorphic genotype may help predict specific phenotypic associations.

In conclusion, our study was the first to evaluate the association of the *IGFBP3*-202A/C (rs2854744) polymorphism with acromegaly in a Chinese population. Further larger and long-term prospective studies are needed to verify whether the polymorphisms affect the hormone level, disease severity, and surgical effect in acromegaly.

## Ethics Statement

This study was carried out in accordance with the recommendations of clinical study guidelines, and the ethics approval was obtained from the Ethics Committee of Beijing Tiantan Hospital, affiliated to Capital Medical University in China (No: KY2014-021-02). All individual participants gave written informed consent in accordance with the Declaration of Helsinki.

## Author Contributions

MG, KC, and HL collected blood samples and acquired medical data used in the study. MG, BZ, and PL analyzed the data and wrote the manuscript. RM, LZ, GZ, and YF performed and supervised the research. GZ, BZ, and XK designed the study. All authors read and approved the final manuscript.

### Conflict of Interest Statement

The authors declare that the research was conducted in the absence of any commercial or financial relationships that could be construed as a potential conflict of interest.
